# Disruption Leads to Methodological and Analytic Innovation in Developmental Sciences: Recommendations for Remote Administration and Dealing With Messy Data

**DOI:** 10.3389/fpsyg.2021.732312

**Published:** 2022-01-04

**Authors:** Sheila Krogh-Jespersen, Leigha A. MacNeill, Erica L. Anderson, Hannah E. Stroup, Emily M. Harriott, Ewa Gut, Abigail Blum, Elveena Fareedi, Kaitlyn M. Fredian, Stephanie L. Wert, Lauren S. Wakschlag, Elizabeth S. Norton

**Affiliations:** ^1^Department of Medical Social Sciences, Feinberg School of Medicine, Northwestern University, Chicago, IL, United States; ^2^Institute for Innovations in Developmental Sciences, Northwestern University, Chicago, IL, United States; ^3^Department of Communication Sciences and Disorders, Northwestern University, Evanston, IL, United States

**Keywords:** developmental methods, remote adaptation, innovation, telepractice, analytic processes, COVID

## Abstract

The COVID-19 pandemic has impacted data collection for longitudinal studies in developmental sciences to an immeasurable extent. Restrictions on conducting in-person standardized assessments have led to disruptive innovation, in which novel methods are applied to increase participant engagement. Here, we focus on remote administration of behavioral assessment. We argue that these innovations in remote assessment should become part of the new standard protocol in developmental sciences to facilitate data collection in populations that may be hard to reach or engage due to burdensome requirements (e.g., multiple in-person assessments). We present a series of adaptations to developmental assessments (e.g., Mullen) and a detailed discussion of data analytic approaches to be applied in the less-than-ideal circumstances encountered during the pandemic-related shutdown (i.e., missing or messy data). Ultimately, these remote approaches actually strengthen the ability to gain insight into developmental populations and foster pragmatic innovation that should result in enduring change.

## Introduction

Child development is characterized by rapid transitions in social-emotional, cognitive, communicative, and motor abilities in the first years of life that are heavily influenced by the environment. Increasingly, the developmental sciences incorporate multi-level methods to most effectively capture intra- and inter-individual differences in developmental pathways. A typical research design utilized in developmental sciences is the longitudinal study in which participants are recruited at a young age, potentially even before birth, and followed across a pre-determined time series in order to gain a rich characterization of their development within a cohort. Study visits may be multiple hours in length, involve intensive measurement (e.g., neuroimaging methods and behavioral observation) and are typically administered within controlled laboratory environments. Given the importance of comprehensive in-person assessment in the developmental sciences, the impact of the COVID-19 pandemic on research cannot be overstated. We argue, however, that this disruption to the status quo of developmental research has provided a unique opportunity for innovation, improved accessibility, and pragmatic application of many measures. The current paper discusses the adaptations to behavioral assessments that were adopted during the COVID-19 shutdown that allowed for continuous data collection in two longitudinal samples when in-person assessment was no longer possible.

*Disruptive innovation* is a concept commonly used in business and marketing to refer to the situation in which a novel technology, strategy, or model surpasses the current seemingly adequate version to attract a new audience or encourage the current audience to increase engagement (Christensen, 1997). Critical to this innovation is that it is not a novel discovery of a new product; for example, Amazon did not invent bookselling – they merely innovated the model for doing so online. Disruptions are not new, as Insel (2009) posited that mental health disorders are neurodevelopmentally unfolding syndromes, which was considered “disruptive insight” to the field of psychiatry. Critical to the theory of disruptive innovation is that established institutions have very little incentive to adopt the new model when the perception is that their current model (e.g., in-person assessment) is successful. Here, we argue that the COVID-19 pandemic has forced developmental science to engage in disruptive innovation in that the model for conducting assessments has changed and it should not return to the previous model once a healthy environment (or as close as possible) is restored.

The field of developmental psychology has been moving toward remote assessment *via* online methods in recent years. Lookit is an online platform in which caregivers can sign up to have their children engage in behavioral studies *via* webcam. Lookit studies typically assess visual attention *via* looking time and preferential looking (for a full review of Lookit, including strengths and limitations, please see Scott and Schulz, 2017a; Scott et al., 2017b). Caregivers can login at their convenience and their child can participate from home if a webcam is accessible to record their child’s responses. Other platforms for unmoderated remote studies include Discoveries Online (Rhodes et al., 2020) and ChildrenHelpingScience.com. Some sites, including ChildrenHelpingScience.com, also provide the opportunity to sign up for appointments with study staff for an interactive experience remotely. Specific research labs have also set up sites, including TheChildLab.org and themusiclab.org, in which families can participate in remote assessments. A recent publication from Sheskin et al. (2020) takes the next step in proposing an online “superlab” to encourage reproducibility by sharing data across studies. Most, if not all, of these platforms support discovery-based experimental paradigms rather than adapt existing gold-standard measures. Here, we will take a focused look at the steps taken to adapt standardized clinical assessments for remote administration *via* a moderated, interactive model.

### An Illustrative Example

Illinois had a critical role in the early timeline of the pandemic spread given that the second identified case of COVID-19 in the United States was from a Chicago woman (Tribune Staff, 2020), and she was involved in the first person-to-person transmission in late January 2020. In mid-March of 2020, all non-essential in-person activities at Northwestern University were suspended, leading many research teams toward a remote work model. This was disruptive to the multitude of developmental science labs located at Northwestern. For the current paper, we will focus on two longitudinal studies being conducted within the Developmental Mechanisms Program at Feinberg School of Medicine and the Institute for Innovations in Developmental Sciences. The NIH-funded “When to Worry” (W2W) studies aim to jointly characterize markers of mental health and language disorder risk across the toddler period. Enrollment is ongoing and the majority of the sample was between 2–3 years of age at the time of the shutdown. Families could participate in the W2W studies if the participating child was between 12 and 18 months for the initial recruitment sample or 24 months of age with a language delay in the language delay sample. One biological parent completed surveys, therefore, eligibility included whether English was spoken at home at least 50% of the time (80% for language delay sample inclusion). The only exclusion criteria were a diagnosis of a developmental or congenital physical disability, or birth before 36 weeks gestation. The Promoting Healthy Brains Project (PHBP) is a randomized clinical trial aiming to use precision medicine approaches to tailor a maternal stress reduction intervention guided by biobehavioral real-time indicators. Women and their infants are followed until the child is 2 years of age. PHBP was enrolling pregnant women in March of 2020. Inclusion criteria include having a gestational age below 22 weeks, planned delivery at a Northwestern-affiliated hospital, ability to complete surveys, assessments, and intervention sessions in English, and specific technology criteria related to the delivery of a prenatal stress intervention (i.e., access to Wi-Fi and a smartphone). Exclusion criteria included pregnancy complications that place infants at risk of neurological disorders or a diagnosis of a severe chromosomal or congenital abnormality in the infant.

Both longitudinal studies included standardized behavioral assessments of the child, computer/tablet-based tasks, parent-child interactions, parent interviews, MRI, EEG, eye-tracking, and parent surveys at various timepoints. While some data collection, for example, survey administration *via* online tools such as REDCap, could continue unaffected, the move to remote working environments jeopardized the ability to collect in-person data, which was central to these studies. We rapidly adapted two behavioral assessments utilized to characterize children’s development for remote administration. The Mullen Scales of Early Learning (Mullen, 1995) is a standardized behavioral assessment of children’s development appropriate from birth to 68 months assessing the following domains: receptive language, expressive language, gross motor, fine motor, and visual reception. The Mullen utilizes a series of prompts/activities often involving manipulatives to engage the child in the behavior of interest. This assessment is often administered in clinical research settings as it provides a *T* score centered on *M* = 50, *SD* = 10, which can be used to determine percentile rank and age equivalencies for each domain. The Mullen is designed to encourage the optimal performance of the child in a research/clinical setting. The Disruptive Behavior-Diagnostic Observation Schedule (DB-DOS; Wakschlag et al., 2008) is a research paradigm relying on behavioral observation that aims to “press” for irritable affect in infants and young children. The DB-DOS examines young children’s emotion regulation capacities and is a valid and reliable tool for distinguishing between normative behavior and clinically concerning disruptive behavior. This assessment utilizes a caregiver context in which the level of support provided by the caregiver on a given task is varied and an experimenter context in which the child interacts with a research assistant with little support from the caregiver. Tasks, often involving manipulatives, are designed to be developmentally appropriate to reflect everyday activities while still placing demands on young children’s regulatory capacities to elicit clinically salient behaviors (e.g., having to wait to play with attractive toys). The DB-DOS was initially designed for preschool-aged children, but a recently adapted version is available for toddlers ages 12-to 18-months. We will outline the steps our research team engaged in to adapt these measures to the stay-at-home orders related to COVID-19.

First, there was recognition that we were not the only ones scrambling. The significance of this pandemic and the related stay-at-home orders were affecting researchers at the global level. The Institute for Innovations in Developmental Sciences at Northwestern organized a sharegroup meeting that bridged developmental labs at both the biomedical and social sciences campuses. Developmental researchers and their teams from across disciplines were invited to present on their remote adaptations to research paradigms, as well as participate in discussions related to complicated issues associated with assumptions regarding participant population resources, including possession of computing equipment with cameras and audio, access to Wi-Fi connectivity, and whether caregivers would have the bandwidth to continue to engage in research at this stressful time. Outreach to Pearson (which publishes many standardized developmental assessments) led to qualified permission to adapt measures for remote administration, ensuring that researchers within this sharegroup were in compliance with legal contracts. This “stronger, together” mindset allowed the researchers to focus on adaptation while institutional resources could focus on regulatory and compliance issues and facilitate nimble transition to the remote environment.

We carefully adapted behavioral assessments to be pragmatic and engaging to meet the needs of our developmental populations while maintaining standardized practices, essentially converting a complex laboratory study into a field study (Glasgow and Riley, 2013; Morris, et al., 2020). Key to our use of the term pragmatic here is the reduction of a lengthy in-person visit to a more concise administration, and critical to success during the pandemic was that the administration could be conducted remotely. The following sections include our recommendations for adaptation and implementation of remote behavioral assessments with infant and child participants (and their caregivers as test administrators/moderators). A central focus of these adaptations was careful consideration of issues related to scientific integrity, measurement validity, and construct continuity with in-person assessments before and after the pandemic.

### The Move to Online: Technological Adaptation

Given that COVID-19 restrictions limited opportunities for in-person assessment in both the lab and home environment, the only alternative for data collection was to move online for remote protocol administration. Northwestern adopted Zoom software for online activities and our ongoing study activities were granted Institutional Review Board approval to collect data *via* this video conferencing platform. Zoom has numerous settings and our research team found the Zoom subreddit^1^ to be instrumental to their awareness of updates and problem-solving issues. This section will discuss challenges and resolutions to issues related to the use of Zoom.

Early in remote protocol development, the research team developed PowerPoint presentations to present to participants on Zoom. The goal was to record children’s behavioral responses to prompts with visual stimuli (e.g., “Can you point to the ball?”). Immediately, the team encountered an obstacle related to screen recording as the Zoom default recording setting did not record the child’s face and instead recorded all audio and the screenshared PowerPoint presentation. To address this issue, the view in Zoom had to be set to “gallery” and the participant’s video set to “pinned” in order to record the child’s behavioral responses and not the screenshared PowerPoint presentation. The team also experienced issues screensharing with an app that was designed to assess executive function, as it was originally screenshared *via* Airplay, but that resulted in frequent audio and video lags. With assistance from the Northwestern’s IT department and Reddit, the research team amended protocols to include a third device (i.e., an iPad) from which to screenshare the app directly, no longer requiring Airplay. This troubleshooting was not limited to visual displays in Zoom.

Our team also had to adjust settings and use a third device to resolve audio issues within Zoom. The DB-DOS requires that an audio clip play during the “crying baby task”.^2^ This audio clip of an infant crying is part of pretense that there is an infant off-screen who is in distress. The goal of this task is to measure how the child reacts to this stressor. The team utilized the “share computer audio” Zoom feature, which played the audio clip in a web browser open on the team’s computer. We found that the quality of the audio was not rendered perfectly *via* Zoom, although this may be due to differences in device speakers and families’ internet speeds. Zoom seemed to have particular trouble projecting the sound of a bell. A ringing bell was used in several tasks as an indicator that time for a task had expired; however, Zoom often dampened this audio to filter out nonspeech sounds, ultimately preventing participants from hearing it. The team switched to the “cosmic” iPhone ringtone as it could be heard clearly *via* Zoom. Although this particular sound was effective for our purposes, we do not recommend the use of Zoom to convey audio information for tasks in which precise sound information is integral to the task’s purpose (e.g., sound discrimination or nonword repetition tasks).

In the beginning months of the pandemic shutdown, many caregivers struggled to use Zoom during remote visits. One critical role that research assistants took responsibility for was providing technological support during these visits, as the platform functioning was essential to ensuring fidelity of administration, accuracy of scoring, and child compliance with the tasks. One method for addressing this issue before a problem was evident was to discuss the caregivers’ comfort level with technology during the visit-scheduling phone calls (e.g., Manning et al., 2020). The research assistants provided support ahead of the visit regarding how to access the Zoom link and what kind of device/setup would be ideal to help alleviate caregiver anxiety and reduce troubleshooting in the moment of the visit. One issue we discovered was related to the participants’ view on their screen during the remote visit. Instead of the gallery view of speakers that is typically presented in Zoom, we wanted the participant to be able to see the PowerPoint slides that had been created to present stimuli (more detail provided below). As such, the research assistant must be cognizant of this issue and remind the caregiver to adjust the “speaker window” as needed to ensure the appropriate stimuli are in view. Young children also struggled when interacting *via* Zoom as they became confused or distracted by the research assistant or their own faces on the screen. These issues were minimized by turning off the participant view in the Zoom visit. We also found that this confusion decreased later in the pandemic, possibly because children and caregivers became more familiar with videoconferencing with family, school, work, and other contexts.

### Preparation for Remote Administration

The first challenge to remote administration was ensuring that we could conduct standardized assessment within a setting in which we had less control (e.g., the participant’s home). First, we adapted our protocols and scheduling scripts to include information about the format of the remote visits. Some items or measures required very specific materials for the child to manipulate, such as a series of cups that nest inside each other, whereas other materials such as a spoon may be available in most homes (see Supplementary Materials for a list of generalized objects). Caregivers were contacted to ensure they were comfortable completing the visit at home and assisting in administration of some items. Many families in the study who had completed lab visits before the pandemic were accustomed to playing a role in measure administration, for example, the caregiver follows a series of written prompts in the caregiver context of the DB-DOS during typical administration. Many caregivers expressed enjoyment in taking on the role of assisting with administering items and appreciated this as an alternative to visiting the lab for those activities.

An immediate challenge was ensuring the families had the necessary items to complete each task in the remote protocol. We created a visit box to send to each family that included the materials needed for the visit (see Supplementary Materials), varying slightly depending on the child’s age, preferences, and special considerations. Each package included a letter to the family explaining the materials in the box and provided more detail about the procedure for the visit, for example, instructions for the administration of the DB-DOS specifically outlining the caregiver’s involvement. In the box, materials for each assessment were placed in separate clear plastic zip-top bags and labeled with the test or activity, item number, and contents. The visit box was shipped to the participant’s home using 2-day shipping with tracking. When explaining the remote protocol, families were asked to avoid opening the visit box until the time of the visit to ensure that materials were not misplaced and to support children’s engagement with these novel items during the visit.

One challenge to the use of a visit box was the cost as it was not planned in our original study budgets. Materials accounted for a large cost as we determined that it would be best for families to keep the items in the visit boxes (e.g., crayons, bubbles, and small toys). Families expressed appreciation for this consideration of health and wellness, as well as the convenience of not needing to orchestrate return shipping. Our team also encountered issues with 2-day shipping, as the visit box was not always delivered within the specified timeframe. If families had not received their visit box, the remote assessment had to be rescheduled. We also had a number of visit boxes go missing during shipping, which meant delaying the study visit date even further to ship a new box. Overall, the visit boxes had some issues but allowed us to provide a standard set of materials to families in our studies.

Immediately prior to the remote study visit, we conducted a “home setup call” with each family. During this call, we asked whether caregivers had issues with internet access or Zoom that they would like to discuss. We asked the family to complete the visit in a small room with no toys present if possible, yet some home layouts did not allow for families to be in a separate room. We also asked that they set the visit for a time when other siblings or pets could be cared for, as study visits were often prolonged when there were multiple distractions present. We adjusted our protocols to build in breaks between assessments that required attention toward the computer screen (e.g., the Mullen) or that relied on caregiver-child engagement during frustrating tasks (i.e., the DB-DOS) so the participant had the opportunity to decompress. Although not every visit had the ideal setting, we were able to prepare the families for the structure of the protocol in advance and make changes to our structure to accommodate the real-world demands of the home study environment.

Although each remote adaptation was designed to be standardized across participants, there were obstacles that made this more challenging. First, the remote assessments were designed to be administered on a computer with participants being recorded (for reliability scoring) *via* the computer’s camera. The use of this technology assumes that the family has a computer in the home, which is not always the case. It was possible to administer the protocol on a smartphone, but this resulted in a decrease in the size of visual stimuli presented and greater difficulty for the research team to code the participant’s responses (e.g., pointing) due to the smaller screen size. Notifications from the phone were sometimes a distraction during study visits. Additional technological limitations for some families included the cost of phone data or plans, varying internet speeds, technological expertise, and unreliable video quality. One possible future solution would be for the research team to loan a computer and/or a cellular hot-spot to a family for the purpose of the study visit. It is also important to consider the assumptions made when conducting remote study visits, including more generally whether the home is a safe place to conduct a visit and whether the research team is trained to respond appropriately if they identify reportable events when conducting a visit. A second assumption is that this protocol is easy for caregivers to administer: future research should examine the caregivers’ perspective with regard to administering these measures with their child. Perhaps this first-person role in the evaluation of their child’s abilities, including cognition, motor, and language skills, is not comfortable or “natural” for them. Understanding this perspective is essential as the field moves in this new direction. We will now discuss the adaptations we made to the Mullen and DB-DOS in more detail.

### Adaptation of Assessment Protocols

All assessment adaptations were discussed in depth with the research team and piloted to determine whether the adaptation resulted in infant/child response data that could be coded prior to implementing the new protocols with research participants. The Mullen Scales of Early Learning is a standardized assessment of children’s gross motor, visual reception, fine motor, expressive language, and receptive language abilities. To be clinically valid, it requires precise administration of a stimuli set with a trained administrator and the child in a controlled environment with very little distraction. As we have outlined above, this is not the ideal assessment for remote administration. However, this assessment was a primary outcome in the W2W study and therefore was critical to adapt for the home environment. The first step in adaptation was to examine the specific item administration for each domain of the Mullen to determine feasibility. We modified the in-person protocol to include which subtests and items were to be completed during the virtual visit as well as the administration order. Caregivers were presented with an introduction that discussed the domains of the Mullen and the expectations regarding their child’s behavior (e.g., “This set of activities is designed to capture a wide range of skills that your child may or may not have just yet.”) The introduction stressed that the assessment needed to be administered in a specific way and asked that the caregiver follow the instructions on the screen as closely as possible. Caregivers were also encouraged to praise their child regardless of their child’s response and to avoid using language like “correct” or “that’s right.”

We focused on 3 domains of the Mullen, listed in the planned order of administration: Receptive Language, Expressive Language, and Visual Reception. Two domains of the Mullen, Gross Motor and Fine Motor, were removed from the protocol due to time constraints and because we had the ability to gather information about motor development *via* online survey (Ages and Stages). Each domain was a separate PowerPoint to allow for flexibility in administration. Each Mullen item had their own slide(s) that could include an instruction prompt, a stimulus (e.g., an image from the Mullen Stimulus book), or a photo of Mullen materials (e.g., a ball, spoon, car, and chair). To reduce administration time and to optimize child compliance and attention, scores from the participant’s Mullen that was conducted the previous year in-person were used to determine which item would start the domain; therefore, we assumed no regression in ability but ensured that children reached a basal. We focus on the Mullen for this section but note that our team made similar adaptations to the Bayley Scales of Infant and Toddler Development (4^th^ edition) for the PHBP study. For this assessment, the Cognitive domain was excluded in its entirety from remote assessment due to complications with administration.

Some items could not be administered remotely and were removed from the assessment protocol. Decisions were made to exclude items when it was determined by a clinical assessment expert and our research team following evidence during piloting that the feasibility of instructing the caregiver to accurately administer the item was low (e.g., too many steps and complicated instructions). Additionally, many materials used in the Mullen are proprietary and we could not provide those to families. Although this was an obvious disadvantage as it relates to data collection, it was a necessity to ensure participant comfort and safety. To our knowledge, this is the first study reporting remote adaptations to standardized cognitive functioning assessments, such as the Mullen and the Bayley, resulting in little empirical guidance for how to produce standardized scores when items are missing. Therefore, raw scores will be used in most analyses. Non-standard assessments (i.e., when items are not administered) will be reviewed by a clinical assessment expert to determine their validity. Further, previous research has used clinically informed imputation methods for generating standardized scores when items are missing (McHenry et al., 2021). Using this approach, we will be able to generate standardized scores for research questions that warrant the standardization. Raw scores from the clinically informed imputed approach will be compared to the non-imputed scores before standardized scores are used in analyses.

Our remote administration protocols relied on screen shared PowerPoint slides that presented the assessment stimuli and prompts for the caregiver. These presentation slides were designed so that they are accurate, clear, consistent, and easy to read. One lesson from piloting was that confusion was reduced when one lengthy slide was divided into two shorter slides. For example, each Mullen item included a slide with instructions and a slide for the item administration that included any necessary prompts and/or stimuli. When a Mullen item required the child to look at and/or point to a picture, the prompts for the caregiver were placed at the top of the PowerPoint slide just above the picture. The researchers had to take extra care to minimize the written instructions or cues for caregivers that offered additional hints to children. For example, one slide listed different colors that the caregiver should ask the child to identify (e.g., “point to the red crayon, point to the blue crayon…”) and the text of the colors matched the prompted color. The researchers realized that while the color coding may aid in clarity for the caregiver, it also provides a hint for the child. As such for this example, the text of the colors was changed to a uniform black. All instructions and prompts were displayed in a “user-friendly” manner, yet wording did not deviate from the Mullen manual. All prompts were typed in bolded font and presented within quotation marks. All actions (such as pointing) were typed in italics. Furthermore, text was consistently located in the same areas of each slide, so the parents were primed to read the instructions and prompts.

Whereas adaptations to the Mullen required that we adhere as strictly as possible to the standardized administration, we were able to adopt a more pragmatic approach when adapting the DB-DOS (Wakschlag et al., 2005, 2008). The DB-DOS is designed to elicit variability in behavioral and emotional (dys)regulation and to provide clinically informative ratings of irritability within the developmental context. Specifically, the DB-DOS uses “presses” to efficiently elicit typical:atypical distinctions in irritability in young children. Because of its objective to examine these patterns across interactional context, the DB-DOS includes presses that occur during interactions between the child with a caregiver and the child with an examiner. Naturalistic presses have ecological validity as they mimic those experienced in children’s daily lives (e.g., the child must wait while the caregiver is engaged in another task). We generated a broader, more flexible DB-DOS paradigm that had a number of pragmatic refinements that still retained essential features. We have termed this pragmatic adaptation of the DB-DOS, the Early Regulation in Context Assessment (ERICA). The ERICA has multiple modes of administration, can be employed beginning at birth, and may be coded *via* a single observation rather than through traditional multiple iterative video passes. Its core feature is the use of developmentally appropriate, ecologically valid presses retained from the DB-DOS, as these have been shown to elicit higher rates of variability than standard observations that do not include presses (Hampton et al., 2020).

To adapt the ERICA for remote administration, the paradigm was shortened from 45 min to 20 min. To achieve this, we prioritized tasks that included presses for multiple domains (e.g., frustration, irritability, and anger). As a meaningful interaction between a young child and examiner was difficult to construct remotely, only the caregiver context was included in the remote adaptation. Presses were adapted to include only tasks that required items feasible and not cost-prohibitive to send in the visit box (e.g., finger paints and bubbles). These pragmatic adaptations have resulted in an improved design for this established behavioral paradigm.

Finally, the research assistant and the caregiver had to work collaboratively over Zoom to administer assessments properly and to manage the child’s behavior. Caregivers were integral to the success of these remote visits, as they did the actual task administration with the child. Research assistants aimed to develop a strong rapport with the caregiver to ensure fidelity of task administration and standardization across families. Written and oral instructions were drafted and revised to ensure clarity while being mindful of maintaining a 6th-grade reading level. Research assistants engaged in partnership building strategies including acknowledging that the protocol could be difficult for the caregiver to administer, praising the caregiver’s effort in following instructed prompts, and emphasizing that the research assistant is available to help answer questions and to chat with the child if the caregiver needed a break. When caregivers showed hesitation or looked uncertain, pauses were enacted to ask if they had any questions regarding how to move forward. Research assistants reported that they felt it was important to meet the caregiver where they were most comfortable with regard to administration feedback. If a caregiver deviated significantly from the instructions (e.g., to the point that the task demand was now different), research assistants paused the task and provided gentle corrections and asked to have the item repeated, often with a slight delay. Deviations were noted in visit notes and flagged for review by a clinical assessment expert. While many caregivers welcomed corrections during administration, some become defensive or more nervous, which became an important area for feedback and growth during our training sessions. We also found that children often lost focus while waiting for the research assistant and caregiver to finish discussing instructions. In response, we implemented planned breaks for the child or we added small animations of animals to the PowerPoint slides during these transitions to keep them engaged. To ensure fidelity of administration and scoring, all assessments were recorded with caregiver permission and sessions were reviewed by a clinical assessment expert.

### What We Lost and What We Gained: A Hybrid Approach

Unfortunately, some methods of data collection were not suitable for remote adaptation, specifically EEG, MRI, and eye-tracking. There are mobile versions of eye-tracking and EEG that were not feasible for our current studies given the shutdown restrictions. As restrictions regarding in-person activities lifted, the realization that we could return to the lab sparked a new focus: Can we optimize the protocol such that some of the study timepoints remained remote while additional new timepoints focused on these missed activities? Decisions had to be made about what was essential to addressing our programmatic research questions. Each study protocol was dissected to determine what assessments were not optimal for administration remotely. For the PHBP, two remote study visits were added: one when the participant was 7–9 months and a follow-up at 2 years. An original 12-month assessment timepoint was maintained with a new design: first, families complete a remote study visit, followed by an in-person visit that includes MRI and EEG, as well as an abbreviated behavioral assessment that includes cognitive and executive function tasks that were difficult to administer online. The W2W study added a timepoint to measure parent-child interaction, parent stress levels, child language, COVID-19 illness, and the overall impact of the COVID-19 pandemic on families’ everyday routines *via* videochat, and online surveys with support from a supplement from NIH. The inclusion of these additional timepoints was facilitated by supplemental grants that aimed to examine families’ experiences during the pandemic. Here, the strength of the disruptive innovation is evident as the study design of incorporating both remote and in-person assessment facilitates rich characterization of families while reducing burden on them.

Our in-person protocols were also adapted to align with health recommendations from the CDC, capacity restrictions from Northwestern, and precautions necessary to keep our staff and participants safe. A pandemic research plan was drafted and submitted for approval by the Feinberg School of Medicine Office for Research. This included safety and health procedures, as well as occupancy limitations and scheduling accommodations to maintain the lowest level of health risk to our staff and participants. Some features of this plan include health screenings of the participants and the staff members conducting the visits 24 h prior to the in-person visit; temperature screenings upon arrival to the study visit; cleaning protocols and ventilation accommodations, including having a HEPA air filter in the study room; adult participants were required to mask and young children were encouraged to wear one throughout the study visit; and staff wore KN95 masks during all visits and were each provided with a face shield. With these safety procedures in place, we still faced hesitancy from participants to complete in-person visits. Some caregivers expressed reassurance in our safety procedures but did not feel comfortable having to take public transportation or ride-share. Additionally, many families faced issues with childcare due to other children being home. Pre-pandemic, we would provide families with childcare in the lab; however, this was eliminated due to capacity and staffing restrictions. For the families that did participate in-person, many caregivers expressed that this visit was one of the only excursions they had taken with their child since the pandemic began. As of June 11, 2021, Chicago has entered into Phase 5 opening (Illinois Department of Public Health, 2021), meaning that restrictions have been fully lifted in nearly all environments, including research settings outside of hospitals/clinics. As we move into this new level of comfort and an increase in in-person activity, it is important to reflect on how the ability to continue to collect data remotely was critical to characterizing the participants and their families during one of the most tumultuous times in recent history.

The move to remote study visits did allow for some opportunities and advantages. For example, *via* remote visits, we could continue to include families that moved out of state during 2019–2020. Previously, their participation would have been limited to survey and phone interviews because most would not be able to travel to the lab for in-person assessment (our study did not budget for long-distance travel). Caregivers commented that it was easier for them to schedule the study visits because of the lack of commute time and the ability to conduct the assessment in their home. Also, providing this remote option helped us gain insight into the development of the child when caregivers were hesitant to come in-person. Children also appeared more comfortable during the remote assessment, possibly due to the familiar setting (e.g., their own snacks to eat and their own bathroom to take bathroom breaks). Whether this comfort then allowed the children to perform at a level that is a more accurate reflection of their skills and knowledge on standardized assessment is an open question for future research. Many of our in-person assessments relied on caregiver-child interaction (e.g., the DB-DOS); as such, the fidelity of those assessments was largely maintained. Standardized assessment, like the Mullen, presented unique challenges, as discussed. We highly recommend video recording of remote assessments, if possible, as this affords the opportunity to ensure fidelity of task administration and scoring *via* review.

### Considerations and Strategies for Handling Missing and Messy Data

Methodological approaches to managing missing data are particularly critical for longitudinal research, as attrition is bound to occur. Although missing data are indeed commonplace in developmental studies, ignoring their presence and impact on study findings can lead to biased results and conclusions (Little and Rubin, 2002; Schafer and Graham, 2002; Jeličić et al., 2009). Arguably, the COVID-19 pandemic has fostered unavoidable and more extreme levels of missingness than what are typical (i.e., more than 50% missing; Enders, 2013), prompting creative problem-solving on the part of the researcher. Further, the pandemic may have introduced more measurement “messiness” or more measurement variability, including less standardization of assessments (e.g., distractions in the home) and collecting aspects of assessments in different ways (e.g., one Bayley scale was collected in person and another remote). In this section, we provide a brief conceptual overview on methods for handling missing and messy data, and practical steps we have taken in our own research for documenting and tracking missingness and changes in methods. We encourage researchers to seek out seminal papers on the topic for further information and guidance (Rubin, 1976; Little and Rubin, 2002; Schafer and Graham, 2002; Jeličić et al., 2009; Enders, 2013; Little et al., 2014).

### Types of Missing Data

Although COVID-19 has exacerbated the issue of missing data in developmental research, these problems of missingness and messiness are not insurmountable. In fact, there are several robust methods for dealing with missing data that allow researchers to draw valid conclusions from the results. Before we determine the method for handling missing data, we must first identify the type of missing data with which we are working (i.e., why these data are missing). According to Rubin (1976), there are three common mechanisms for missing data. Data are considered missing completely at random (MCAR) if the probability of missing data on a given variable is unrelated to the other measured variables. As an example, in order for our data on irritability to be considered MCAR, we would have to find that no measured variables in our study predicted whether an infant had missing irritability data. Data that are missing at random (MAR) are those that are related to variables other than the variable with missing data. In other words, data are MAR when the missingness is a result of other measured variables. Continuing with the same example, the data would be MNAR if missing rates for irritability were related to another variable in our study (e.g., harsh parenting), but not related to irritability. Finally, data are considered missing not at random (MNAR) when the probability of missing data on a variable potentially depends on the missing value itself. So using the current example, despite controlling for our other measured variables, infants high in irritability would be more likely to have missing values for irritability. Pauses in data collection due to the COVID-19 stay-at-home order may initially seem to be a source of MNAR, but participants with data gaps due to COVID-19 may not necessarily differ in a systematic way from those without these gaps (i.e., those individuals who visited the lab before the order was in place). Once the reason for missingness is assumed (given that some assumptions of MAR and MNAR are unable to be directly tested), researchers should report it in their manuscript, as well as the methodological rationale for handling the data (Enders, 2013).

### Best Practices and Statistical Methods for Handling Missing and Messy Data

#### Documentation for Sensitivity Analyses

Documenting and tracking reasons for missingness in close proximity to the data collection process allows for more sophisticated missingness analyses once data collection is complete. Including questions about the status of data collection to track missingness and deviations from the original protocol can be used to derive variables for potential model parameters. Throughout the pandemic, we have recorded dates for suspension and resuming of in-person activity. From these data, we can construct a variable to differentiate participants who withdrew from the study from those who were physically prevented from providing data due to restrictions or government mandates on visits. Further, given our rapid response to changing method administration to continue collecting data, for any measures that vary in their mode of data collection (i.e., were administered remotely or in-person), we have created a field in our database to document which method applied to that individual visit. Sensitivity analyses can then be used to address these patterns of attrition and changing methods. As an example, we can examine whether scores on the Mullen vary by collection method (fully in-person vs. remote). First, we can create a variable for collection method by dummy coding the method of administration (e.g., 0 = in-person; 1 = remote). Then, we can use this variable to determine whether Mullen scores vary by collection method. If Mullen scores do not vary by collection method, then statistical analyses can proceed as planned. These dummy-coded variables should also be considered for inclusion in the main study models as controls if there is theoretical justification (e.g., if the researcher would expect the outcome to change depending on method of collection). With respect to repeated measures data, we can take a missing modeling approach to test the most frequent occurrences of patterns of missingness. For example, we might find that missing the second measurement occasion is the most frequent type of pattern, or overall, we are finding five common patterns of missingness that apply to most of our sample. Again, we can dummy code these patterns and include them in a model. In a growth curve analysis, we can test whether missingness patterns affect the intercept or slope of our construct of interest over time. We may find that these patterns of missingness do not influence trajectories, and again, we can proceed as planned. Documenting dates during which measurement occasions occurred can also allow for a continuous time metric, for which we can model trajectories for the participants (D. Mroczek, E. Graham, & E. Beck, personal communication, December 09, 2020).

#### Statistical Methods

Multiple imputation and full information maximum likelihood (FIML) are two popular and robust methods for handling missing data that follow MCAR or MAR assumptions (Jeličić et al., 2009; Little et al., 2014), both of which we plan to leverage in our data analysis. Multiple imputation is the process of copying the original dataset to generate multiple datasets that fill in missing values with plausible estimates (Rubin, 1987). By using this method, the values are maintained in the datasets to prepare them for analysis. The analysis is then fitted on the imputed datasets and pooled estimates are derived. By creating multiple datasets, variability is increased and the findings are arguably more generalizable than if one were to rely on a single imputation (Jeličić et al., 2009). To produce this needed variation, 20 to 100 imputations are likely sufficient (Graham et al., 2007). Auxiliary variables, or those variables that are related to the variables with missing data, should be specified in the imputation to correct for some biases inherent to the nonresponse (Schafer, 1997). Multiple imputation methods are available in many statistical software programs.

FIML, by contrast, imputes missing data for deriving model estimates, but then deletes the imputed values after the analysis is complete. Thus, FIML will not produce a dataset with imputed values as multiple imputation does. FIML uses the data from partially completed variables to estimate parameters. In this way, linear relations between the missing data variable and the other variables in the model work to generate the estimates (Little and Rubin, 2002; Schafer and Graham, 2002). Many software packages are able to implement FIML, and for some modeling techniques, it is the default strategy (e.g., growth curve modeling; Enders, 2013). Both multiple imputation and FIML are widely used methods for managing missing data, but in some cases, one method may be preferred over another. For instance, FIML may be more appropriate when the dependent variable is incomplete, whereas multiple imputation does not distinguish between independent and dependent variables in the imputation process. FIML often requires that the distribution of the variable with missing data be multivariate normal, whereas multiple imputation is less rigid (see Enders, 2013 for a review).

Although less frequently used, a Bayesian modeling approach can be applied for handling missing data. As mentioned, MAR and MNAR are assumptions and cannot be formally tested. Bayesian modeling can formalize these more subjective assumptions (Daniels and Hogan, 2008). With a Bayesian analysis, the imputation model and the analysis model are fitted at the same time, whereby estimates are acquired from posterior distributions of the parameters and missing variables (Ma and Chen, 2018). However, this approach is typically not recommended if one does not have prior experience with Bayesian modeling.

We have overviewed several potential methods for statistically handling missing data, but there are two notably flawed methods that should be avoided (Little and Rubin, 2002). Listwise deletion is the process of deleting cases that have missing values for all analyses, and pairwise deletion is the process of deleting cases depending on the analysis. Because both methods eliminate incomplete cases, the analysis has less power. Further, removal of cases because they are missing may introduce biases to the findings (Enders, 2013). Importantly, the appropriate method for handling missing data depends on the specific data and model in question. As mentioned, these methods require that the data meet several assumptions and depend on what percentage of the data are missing, causes of missingness, and patterns of missingness (Scheffer, 2002; Jeličić et al., 2009). The percentages of missing data for each study variable should be reported, regardless of which missing data method was used. Further, there may be added complexities with particular data types. For instance, researchers have debated how to handle missing neuroimaging data and whether and how these data should be imputed (Matta et al., 2018). However, when we properly track missing and messy data, we can learn to embrace the disruption that is so characteristic to our line of study. Rather than delete these cases, modern statistical approaches and thorough documentation can make up for lost ground and allow us to draw valid conclusions from our findings. We anticipate that we will be able to leverage multiple imputation and FIML techniques with the majority of our data.

### Testing the Predictive Utility of Disruptive Innovative

An advantage of disruptive innovative is that we can test empirical questions about the predictive ability of our new methodology. A first question we can ask relates to the comparability of our methods, such as whether the remote version of our instrument measures the same underlying construct as the version performed in the lab. In the COVID-19 pandemic, it was not possible to collect both in-person and remote measures from each participant, hence the reason for the transition to remote assessment in the first place. However, given the innovation that has stemmed from these unprecedented circumstances, it would be valuable for future work to administer both versions of the measures to formally test their agreement.

Another question we would want to examine is whether our remote methods have predictive utility over more simplistic measures, such as surveys. For example, is it worth the burden to both the participant and the researcher to collect a remote measure of responsive parenting when a survey measure of responsive parenting might suffice? For parenting researchers, the resounding answer may be “yes,” but it is important to empirically test whether our remote measures hold predictive value for our outcomes of interest, particularly when measures may be more intensive. In a new study we have underway (Luby et al., 2019), we are seeking to answer this question by developing a risk calculator for generalizable risk prediction of preschool psychopathology. We argue that although multiple levels of analysis allow us to identify comprehensive risk for psychopathology, assessments at every level for every child may not be feasible and may be challenging to translate to real-world practice. Risk prediction algorithms, in particular, necessitate the inclusion of more intensive or burdensome measures when they add substantial value to the predictive model (Lloyd-Jones, 2010). The goal of our study is to test whether more cost- and resource-intensive measures (e.g., MRI, EEG, and behavior) have greater predictive utility of mental health prediction over less burdensome measures (e.g., survey). Further, the methods needed to predict mental health outcomes may depend on the level of risk for the individual child. For example, using the stoplight metaphor (Smith et al., 2018), children at high clinical risk (red) may receive immediate referral for treatment or prevention/intervention, children at low clinical risk (green) may only receive later testing at their regular well-child visit, and children with high clinical uncertainty (yellow) may require the more intensive measures to more accurately predict risk.

To empirically test the added value of these intensive measures, we can employ three key statistics: concordance (*c*) statistic, discrimination slope, and model calibration. The *c*-statistic is the most common statistic for discriminating risk calculator performance, representing the receiver operating characteristic curve (ROC) (AUC; D’Agostino et al., 1997). The AUC, ranging from 0 to 1, reflects the ability of the risk score to distinguish between having the disorder and not having the disorder. The discrimination slope indicates model improvement in sensitivity and specificity (Pencina et al., 2008). Lastly, calibration measures how closely the predicted probability aligns with real experience (D’Agostino et al., 1997). Using these statistics, we can determine whether a model including more intensive measures can better distinguish between disorder and no disorder than a survey-only model. In sum, by determining which indicators and methods are needed to best predict mental health, we can accelerate clinical translation to prevent disorder onset while limiting assessment burden for both the participant and the researcher.

## Discussion

Given this disruptive effect of the pandemic, what changes in developmental research are likely to endure in a “post-COVID” world? Here we argue, it should not be a return to “business as usual.” While often through this adaptation process our research team felt as if there was no perfect solution, we did determine the optimal settings to conduct behavioral assessments with varying demands on the caregiver and child to support data collection during a global pandemic. Importantly, we plan to continue to use remote assessment protocols in future studies as we found this disruptive innovative to be critical to successful engagement with our research participants and see the potential for this to impact data collection more broadly in the field of developmental science.

Employing hybrid or fully remote research paradigms has great potential to improve representation in research. Typical, lab-based developmental science studies are more likely to engage Western, Educated, Industrialized, Rich, and Democratic (WEIRD) participants from a close geographic area, given that participation is often more accessible and convenient for such families (Sugden and Moulson, 2015; Nielsen et al., 2017). Because development is shaped by early experiences rooted in culture and other features of the environment (Greenough et al., 2002), it is unlikely that many developmental processes are truly invariant across sociodemographic and sociocultural groups, so engaging diverse participants is critical. Importantly, including diverse participants increases the generalizability of research findings (Hammer, 2011; Rowley and Camacho, 2015). In the current set of studies, English proficiency was an inclusion criterion for eligibility and all measures were designed for administration in English. One consideration is the requirement for caregivers to be literate in English given that instructions were provided in a letter included in the visit box and presented on the computer screen. When designing for remote administration, care should be taken to ensure that the demands of the tasks being administered comply with inclusion criteria and do not tax the participant excessively.

There are multiple reasons why diverse families may be less likely to engage and remain in traditional research studies, which new methods may address. Individuals from groups who have been disproportionately mistreated in research in the past, as is the case for Black Americans (Green et al., 1997), may have greater distrust of researchers and be less likely to engage in research, particularly in-person studies. For low-SES and urban caregivers, completion of study or intervention visits is hindered by availability of adequate transportation, child care, and timing of visits during working hours (Gross et al., 2001). Additionally, as we noted previously, we preferred presenting images on a computer screen compared to a smartphone screen for a number of reasons, including improving visibility. This is a limiting factor for participation, although we discuss the possibility of providing loaner computers, which should be considered when determining the feasibility of remote administration of measures. Beyond just recruitment and administration issues, retention for longitudinal studies with many visits over long periods of time can be more challenging for families facing economic hardship, due to frequent residential mobility and changes in contact information that may be more prevalent (Knight et al., 2009) and preclude study completion. Platforms such as Lookit (Scott and Schulz, 2017a) have transformed researchers’ ability to collect data that was previously only possible in the lab. The benefits of offering remote studies that families can complete in their homes, at times convenient for them, may result in greater representation in research through increased opportunity for engagement for nearly all families.

Another important theme for developmental scientists to consider as we move past the pandemic is what research measures are “good enough” to answer the questions of interest (Blackwell et al., 2020; Morris et al., 2020). Whereas a study may have previously collected an in-depth lab-based assessment designed to measure a specific construct, the pandemic has forced researchers to reconsider whether a shortened, remote, or less burdensome method (e.g., a questionnaire) can fill that position (e.g., Manning et al., 2020). This will be an important theme moving forward, as what is most pragmatic or efficient has long been ignored in many developmental studies in favor of what is most in-depth. Pragmatic measures are certainly the future of developmental assessment, given the success of the National Institutes of Health (NIH) Toolbox with children and adults, and its upcoming extension version that covers infancy through early childhood. In its current version, assessment domains, including Language and Executive Function, require approximately 10 min each to administer, with scoring completed on an iPad.

Overall, the COVID-19 pandemic has led to disruptive innovation in methods for remote assessment that will transform research and practice for the better. Efforts range from reimagining and redeploying widely used measures, such as a “mobile” version of the NIH Toolbox (Weintraub et al., 2013), to researchers first considering designing studies to be remote assessment rather than defaulting to in-lab work. Remote data collection also allows unprecedented abilities to collaborate and collect data globally. It is our hope that the scientific and practical challenges that researchers faced during the pandemic will ultimately result in a field that is better equipped to address developmental science questions and provide innovative insights.

## Author Contributions

SK-J, LW, and EN were responsible for drafting and revising this manuscript and oversaw the research adaptations discussed, in collaboration with EA. LM contributed the analytic innovation section. HS, EH, EG, AB, EF, KF, and SW contributed to sections on the adaptions of the methods. All authors contributed to the article and approved the submitted version.

## Funding

Promoting Healthy Brain Development *via* Prenatal Stress Reduction: An Innovative Precision Medicine RCT Approach (Lurie Children’s Hospital of Chicago; Wakschlag), R01MH107652 (Wakschlag), R01DC016273 and R01DC016273-A1S1 (Norton/Wakschlag), R34DA050266-S1 (Wakschlag). The content is solely the responsibility of the authors and does not necessarily represent the official views of the National Institutes of Health.

## Conflict of Interest

The authors declare that the research was conducted in the absence of any commercial or financial relationships that could be construed as a potential conflict of interest.

## Publisher’s Note

All claims expressed in this article are solely those of the authors and do not necessarily represent those of their affiliated organizations, or those of the publisher, the editors and the reviewers. Any product that may be evaluated in this article, or claim that may be made by its manufacturer, is not guaranteed or endorsed by the publisher.

## Footnotes

^1^ http://reddit.com/r/Zoom

^2^ https://www.youtube.com/watch?v=oL2B-AAnsHo

## Supplementary Material

The Supplementary Material for this article can be found online at: https://www.frontiersin.org/articles/10.3389/fpsyg.2021. 732312/full#supplementary-material
