# Supplementary material for: Disruption Leads to Methodological and Analytic Innovation in Developmental Sciences: Recommendations for Remote Administration and Dealing With Messy Data
Source: Front Psychol. 2022 Jan 4;12:732312. doi: 10.3389/fpsyg.2021.732312 (PMC8764157; doi:10.3389/fpsyg.2021.732312)
Supplement: Supplementary file 1 [file Table_1.docx]

## Supplemental Materials

## **Promoting Healthy Brains Project (PHBP) Study:**

## Visit Box Packing List for 7-9 Month Remote Assessment:

1. Welcome letter & caregiver instructions
2. Stacking cups
3. 1 tube of bath paint
4. Baby wipes
5. 4 free play toys
6. 1 rattle
7. 3 pieces of blank paper
8. 2 crayons
9. 1 squeeze toy

## Visit Box Packing List for 12 Month Remote Assessment:

1. Welcome letter & caregiver instructions
2. Stacking cups
3. 1 bottle of bubbles
4. Pudding, 2 tubes of food coloring, and 1 smock
5. 4 Baby wipes
6. 4 Free play toys
7. 1 rattle
8. 3 pieces of blank paper
9. 2 crayons
10. 1 squeeze toy

## Generalizable Items Provided by the Caregiver:

1. 2 small containers
2. 1 mirror or front-facing camera on a phone
3. 5 toys or objects that the infant likes to play with/teethe
4. 1 spoon
5. 1 table or high chair with table attachment
6. 1 cup
7. Food pellets that the infant can pick up (e.g., cheerios, puffs)
8. 1 small ball or other suitable object to throw (can be a crumpled piece of paper)

**When to Worry (W2W) Study:**

## Visit Box Packing List for 24 Month Remote Assessment:

1. Welcome letter & caregiver instructions
2. 1 puzzle
3. 1 bottle of bubbles
4. 1 piece of white paper and 1 crayon
5. 4 small washable finger paints, 4 pieces of white paper
6. Baby wipes
7. 5 finger puppets
8. 1 board book
9. 1 small rubber duck
10. 4 pretend coins
11. 8 crayons
12. 1 piece of gift wrap

## Visit Box Packing List for 36 Month Remote Assessment:

1. Welcome letter & caregiver instructions
2. 1 box of 64 crayons
3. 1 small coloring book
4. 5 finger puppets
5. 1 paddle ball game
6. 1 ball
7. 2 small gift bags
8. 1 bottle of bubbles
9. 4 pretend coins
10. 8 crayons
11. 1 piece of gift wrap

## Generalizable Items Provided by the Caregiver:

1. 2-3 familiar basic toys
2. 1 drinking cup
3. 1 smaller cup
4. 2 identical washcloths
5. 1 teddy bear figurine (or substitute)
6. 1 ring (or substitute)
7. 1 toy car (or substitute)
8. 1 hardcover book (with pictures)
